# Design and Fabrication of Electrospun PLA-Based Silica-Modified Composite Nanofibers with Antibacterial Properties for Perspective Wound Treatment

**DOI:** 10.3390/polym15173500

**Published:** 2023-08-22

**Authors:** Kateryna Filatova, Eva Domincova Bergerova, Natalia Kazantseva, Milan Masar, Pavol Suly, Tomas Sopik, Jaroslav Cisar, Silvie Durpekova, Vladimir Sedlarik

**Affiliations:** 1 Centre of Polymer Systems, University Institute, Tomas Bata University in Zlin, Tr. T. Bati 5678, 76001 Zlin, Czech Republic; 2 Faculty of Technology, Tomas Bata University in Zlin, Vavreckova 5669, 76001 Zlin, Czech Republic

**Keywords:** silica nanoparticles, nanofibers, electrospinning

## Abstract

The aim of this study was to develop a novel amikacin (AMI) delivery system with prolonged release based on composite electrospun nanofibers of PLA supplemented with AMI-loaded Si nanoparticles of different morphology. The resultant materials were characterized in terms of their physical properties (scanning electron microscopy, Brunauer–Emmett–Teller analysis, thermogravimetric analysis, water contact angle). High-Performance Liquid Chromatography was used to determine the AMI content in the liquid fractions obtained from the release study. The results show that nanofibers of fumed silica exhibited an aggregated, highly porous structure, whereas nanofibers of mesoporous silica had a spherical morphology. Both silica nanoparticles had a significant effect on the hydrophilic properties of PLA nanofiber surfaces. The liquid fractions were investigated to gauge the encapsulation efficiency (EE) and loading efficiency (LE) of AMI, demonstrating 66% EE and 52% LE for nanofibers of fumed silica compared to nanofibers of mesoporous silica nanoparticles (52% EE and 12.7% LE). The antibacterial activity of the AMI-loaded nanofibers was determined by the Kirby–Bauer Method. These results demonstrated that the PLA-based silica nanofibers effectively enhanced the antibacterial properties against the *Staphylococcus aureus*, *Escherichia coli*, *Pseudomonas aeruginosa*, and *Klebsiella pneumoniae*.

## 1. Introduction

Treating and managing wounds is largely influenced by the type of dressing applied, with various therapeutic possibilities, including protection of the surface, drug delivery, and antibacterial activity [1,2,3]. Electrospun nanofibers have been widely used in various fields of medicine as dressings for wound healing due to their high porosity, good mechanical properties, and excellent biocompatibility [4,5]. Several techniques for fabricating nanofibers have been developed, examples being phase separation, template synthesis, solvent casting, molecular self-assembly, and electrospinning [6,7]. Producing functional polymeric nanofibers by electrospinning has attracted attention in the past decade for the inherent simplicity of the process, the capacity to enhance the properties of such fibres, and the potential to combine various polymeric materials that differ in structure and morphology [1,3]. This has resulted in fibrous matrices being developed that possess complex frameworks [8,9] of different drug loading capacities [10,11], with a managed distribution of diameters [12,13], thereby expediting a low initial burst and subsequent controlled release of the medication, in addition to heightened antibacterial properties. The greatest challenge faced in devising an electrospun system for drug delivery concerns leveraging control over the high initial burst release typically associated with the properties of such matrices [1]. A dramatic release like this, caused by the high specific surface area of electrospun fibres, is particularly problematic if the given therapeutic agent is a small hydrophilic drug or high doses of it are required [5,10,14]. An approach that helps enable sustained drug release involves fabricating composite fibres which hold drug-containing nanoparticles embedded in electrospun polymer fibres [15,16].

An assortment of biodegradable polymers has been utilized to obtain electrospun nanofibers, e.g., poly(ε-caprolactone) [17], poly(lactic acid) (PLA) [2,18], poly(glycolic acid) [19,20], poly(3-hydroxybutyrate—co-3-hydroxyvalerate) [9,21] and a copolymer made from lactic and glycolic acids [11]. When choosing a polymer, however, consideration should be given to its level of environmental friendliness and sustainability [22,23]. A notable example in this regard is PLA, which is a thermoplastic aliphatic polyester derived from renewable resources, so it constitutes an environmentally friendly, non-toxic, biodegradable polymer [24,25,26]. PLA nanofibers are characterized by favourable processability, easily controlled degradation, good mechanical properties, and high biocompatibility [27], in addition to approval by the United States Food and Drug Administration (FDA) as solutions for human therapy [23,28]. A drawback is that the hydrophobic structure and surface properties of PLA prove difficult to change. Hence it has a low affinity for cells in biomedical applications and sometimes induces an inflammatory response in surrounding tissue upon direct contact [29,30,31]. Various methods can be employed to overcome this problem and enhance the properties of nanofibers, though. One example is the fabrication of biocomposite fibres by modifying the surface of electrospun PLA with organic or inorganic materials. Examples of drug-loaded nanomaterials that have been fixed within electrospun fibres in previous studies are inorganic and polymeric nanoparticles [6,20,32,33], nanotubes [8,34], micelles [35] and liposomes [36,37].

Silica nanoparticles constitute inorganic material of interest to have been incorporated in organic polymer nanofibers [38]. The associated advantages include high surface area, substantial pore volumes, controllable pore sizes, biocompatibility, and the potential for introduction of organic functional groups, as well as possession of GRAS status conferred by the FDA [39]. Other benefits comprise possible synthesis with uniform and adjustable particle sizes, surface chemistry that facilitates physical and chemical modification, and the properties of biodegradability and generally non-toxicity [21,40]. A hypothesis of the authors was that the presence of silica nanoparticles would enhance the hydrophilic properties of PLA nanofibers, a crucial aspect when a highly hydrophobic therapeutic agent is involved and/or substantial loading is necessary. The literature reports that encapsulation of silica nanoparticles heightens the stability of the loaded medication during the electrospinning treatment, which is especially important for thermolabile drug molecules [8,20,41]. Their incorporation helps overcome the greatest challenge in developing an electrospun, drug-loaded system, i.e., establishing control over the high initial burst release typically associated with such matrices. In addition, the antibacterial properties of silica-based materials not only make it possible to enhance such ability but also to administer a reduced concentration of the antibiotic, thus diminishing the occurrence of adverse reactions [20,32].

The aim of this study was to develop an amikacin (AMI) delivery system with prolonged, tailored release kinetics, one based on composite electrospun nanofibers of PLA supplemented with AMI-loaded silica nanoparticles of different morphology.

AMI is a semisynthetic aminoglycoside antibiotic with broad therapeutic action against Gram-negative bacteria. However, AMI is known to cause adverse effects, including nephrotoxicity and ototoxicity [42]. Another disadvantage is poor absorption when administered orally due to the polycationic nature of the molecule. For these reasons, medication with AMI must be a strictly controlled process [43].

In this work, two types of Si nanoparticles, commercially available fumed silica (SIAM) and fabricated mesoporous silica (SIMES), were investigated for their suitability as supports for AMI immobilization in PLA matrice and for efficient production of PLA-based silica-modified composite nanofibers with optimal diameters. As far as the authors were aware, no study had taken place on the impact exerted by the varied morphology of Si nanoparticles on the morphology of nanofibers in connection with an immobilized bioactive compound in such nanoparticles. The authors researched the hydrophobic properties of the nanofibers, the time-dependent delivery of AMI under simulated physiological conditions, and its antibacterial activity against Gram-positive and Gram-negative bacterial strains in order to gauge the applicability of the nanofibers for wound healing.

## 2. Materials and Methods

### 2.1. Materials

The following chemicals were utilized in experiments: amikacin disulfate (AMI; Interquim); L-lactic acid (LA; 80% water solution, Merck Milipore); Tin(II) 2-ethylhexanoate (Sn(Oct)2; ~95%; Sigma-Aldrich, Schnelldorf, Germany,); solvents—chloroform, acetone, methanol (MeOH) and ethanol (all analytical-grade, Sigma-Aldrich, Schnelldorf, Germany); chloroform (HPLC-grade; Chromspec, Prague, Czech Republic); Fumed silica (SIAM; mean particle size 30 nm; mean pore size 6 nm; Sigma-Aldrich, Schnelldorf, Germany); Tetraethyl orthosilicate (TEOS; 98%; Sigma-Aldrich, Schnelldorf, Germany); ammonia (25%), dimethylformamide (Merck Millipore, Darmstadt, Germany) and ultrapure water—utilized as precursor materials for the synthesis of mesoporous silica nanoparticles; and diiodomethane (Sigma-Aldrich, Schnelldorf, Germany).

Antibacterial testing was performed on the following bacterial strains (Tomas Bata University in Zlin, Czech Republic): *S. aureus* CCM 4516; *E. coli* CCM 4517; *Enterococcus faecalis* CCM 3956; *K. pneumoniae* CCM 4415; and *P. aeruginosa* CM 1961.

### 2.2. PLA Synthesis

This was carried out in accordance with a procedure derived from a method described by Pavelkova et al. [44]. In brief, 100 mL of the LA monomer was poured into a two-neck round-bottom flask connected to a condenser. Formulation of the mixture involved a dehydration phase under stirring, at the pressure of 20 kPa for 4 h and in an oil bath at 160 °C, with 0.5% *w*/*w* of Sn(Oct)_2_ added in afterward. The pressure was subsequently reduced to 3 kPa, upon which the reaction continued for 24 h. The product of the polycondensation reaction was precipitated with MeOH and water, filtered off, washed with water and MeOH (10:1), and subsequently dried at 30 °C for 48 h in a vacuum oven (10 kPa). The resultant PLA was characterized by GPC analysis (PL-GPC 220 chromatographic system, Agilent, Santa Clara, CA, USA) as follows: three connected LC columns were used—PL gel MIXED-A (300 × 7.8 mm, 20 μm) + MIXED-B (300 × 7.8 mm, 10 μm) + MIXED-D (300 × 7.8 mm, 5 μm); THF was applied as a mobile phase at the flow rate of 1 mL/min; separation was carried out at 40 °C and the injection volume equaled 100 μL. A refractive index detector and a viscometric detector were employed for the detection. The calibration curves were plotted according to polystyrene standards (580 ÷ 6,000,000 g/mol, Polymer Laboratories Ltd., UK). The average molar mass or molecular weight (*Mw*), number average molar mass (*Mn*), and polydispersity index (*Ð* = *Mw*/*Mn*) of the tested samples were determined from peaks corresponding to the polymer fraction in accordance with the universal calibration method. All data was processed in Cirrus software (Agilent Technologies, Santa Clara, CA, USA). Table 1 summarizes the GPC results for the synthesized PLA.

### 2.3. Synthesis of the Mesoporous Silica Nanoparticles (SIMES)

Preparation of SIMES required that the molar composition of the precursors was maintained thus: TEOS: 0.93, NH_3_: 26, C_2_H_5_OH: 12, H_2_O. A typical process took place [45], whereby 74 mL of C_2_H_5_OH in 10.4 mL H_2_O was stirred, into which 3.1 mL of NH_3_ was added for each set of experiments. This clear solution was supplemented dropwise with 11 mL of TEOS under stirring (400 rpm), and stirring continued for another 4 h at room temperature (25 °C). The resultant solid product was centrifuged (14,000 rpm) and washed, followed by air drying at 60 °C overnight. Finally, the dried powder was calcined at 550 °C for 6 h at a heating rate of 1 °C min^−1^.

### 2.4. Immobilization of AMI in the Silica Nanoparticles

Two types of AMI-loaded nanoparticles formed of either commercially available fumed silica SIAM or fabricated mesoporous silica SIMES were synthesized by the sol-gel method to expedite sustained drug delivery. Incorporating the antibiotic in SIMES and SIAM involved dispersing 500 mg of nanoparticles in 500 mL of AMI aqueous solution (50 mg/mL), and this was subjected to magnetic stirring overnight. The suspension was then centrifuged, and the isolated AMI-loaded silica nanoparticles (SIAM-AMI and SIMES-AMI) were freeze-dried for 48 h. The concentration of AMI in a solid fraction was established by the difference between the initial loading and concentration in the washing solution that was established by HPLC.

### 2.5. Electrospinning

Electrospun nanofibers denoted as PLA-SIAM-AMI and PLA-SIMES-AMI were produced from PLA that incorporated the above-mentioned SIMES-AMI and SIAM-AMI (pre-loaded silica nanoparticles with AMI) at a ratio of 1:1 *w*/*w*. Initial quantities of the compounds in the electrospinning mixture are described in Table 2.

The procedure for this commenced with preparing all the polymeric solutions by dissolving the PLA in a mixture of chloroform and dimethylformamide (4:1 *v*/*v* ratio). In order to fabricate fibres that contained AMI, the drug in powder form was dispersed in a polymeric solution that underwent magnetic stirring overnight so as to homogenize the Si nanoparticles in the polymer matrix solution. The final concentration of AMI in resultant nanofibers PLA-SIAM-AMI and PLA-SIMES-AMI were 3.9 and 4.8%, respectively (see Table 2). For the sake of comparison, PLA fibres containing an even amount of AMI incorporated were also prepared, and the fibres obtained from these formulations were designated as PLA-AMI. The composition of PLA-AMI electrospun formulations is presented in Table 2. As for the electrospinning process, each mixture was spun at a feed rate of 0.5 mL/h under an applied voltage of 60 kV. The distance from the tip of the needle to the collector was set to 20 cm, and a flat aluminum plate was fixed in place to catch stray nanofibers. All related experiments were carried out under ambient conditions.

### 2.6. Elemental Analysis

Energy dispersion X-ray spectrophotometry (EDX-RF) was used to determine the Si amounts in prepared nanofibers samples. The EDX is based on X-Ray analysis. The samples were analysed in powder form by using the ARL Quant’EDX-RF analyser of Thermo-Fischer Scientific. The analysed samples were placed in a Teflon cup (approximately 3 g) and suitably closed with a special microcellulose film, after which they were inserted into the autosampler. Each sample was analysed twice. Samples were evaluated using the chosen method: Any Sample Helium in the UniQuantX program. The software then evaluated the presence and amount of elements and basic matrix (CHON) in mass percent (% m/m).

### 2.7. Water Contact Angles

A SEE System, by Advex Instruments (Brno, Czech Republic), was employed to gauge the contact angles of samples by the sessile drop method, with the aid of 10.5 cm^2^ fibre strips taped to a glass slide. Deionized water, ethylene glycol, and diiodomethane at the volume of 2 μL were utilized as testing liquids for the nanofibers. Mean values for contact angle were calculated from 10 separate readings for each such liquid.

### 2.8. Thermogravimetric Analysis

The thermal behaviour was studied using thermogravimetric analysis on a Mettler-Toledo TGA/SDTA 851e instrument (Columbus, OH, USA) under nitrogen flow (20 mL min^−1^). A heating rate of 10 °C min^−1^ was applied at intervals across a temperature range of 30 °C to 600 °C. The weight of samples varied between 10 and 12 mg. Values for the organic phase in the carriers were determined by taking into account the extent of weight reduction.

### 2.9. Brunauer–Emmett–Teller Analysis

The specific surface area and pore size distribution of the silica nanoparticles and those loaded with AMI were derived from recordings of nitrogen adsorption–desorption isotherms made on a Micrometrics Brunauer–Emmett–Teller (BET)-surface device (Belsorp-mini II, BEL Japan, Inc.). The pore size distribution was ascertained from the desorption branches of the isotherms by following the Barrett–Joyner–Halenda (BJH) method.

### 2.10. Scanning Electron Microscopy (SEM)

SEM micrographs of the prepared formulations coated with a sputtered gold/palladium layer (SC7620 Mini Sputter Coater, Quorum Technologies, Lewes, UK) were obtained on Nova NanoSEM 450 scanning electron microscope set to 5 kV. Mean values for fibre diameter and related distribution were estimated from SEM images, facilitated by measuring the diameters of fifty randomly selected nanofibers with the aid of image analysis software (ImageJ 1.46r; 2012).

### 2.11. In Vitro Drug Release Studies

The release kinetics of AMI were determined by applying a phosphate-buffered saline solution (PBS, pH 7.4) as the release medium. Samples measuring 14 cm^2^ were immersed in 10 mL of PBS in capped glass flasks and then incubated at 37 °C in a thermostatically controlled orbital shaker set to 100 rpm. At predefined time intervals, 1 mL of the liquid fraction of each sample was collected, and the rest of release medium was completely removed and substituted with the same amount of fresh PBS. Sink condition tests were carried out, and the AMI content of each sample was determined by High-Performance Liquid Chromatography (HPLC) described below. Following the release studies, the total amount of AMI present was calculated by dissolving samples in 1 mL of chloroform, and any remaining AMI was extracted by PBS. Release studies were performed in triplicate for each type of fibre.

### 2.12. High-Performance Liquid Chromatography

AMI was determined by in-needle derivatization with o-phthaldialdehyde (OPA) reagent and FLD detection (HPLC Dionex UltiMate 3000 Series, Thermo Fisher Scientific, Waltham, MA, USA). In-needle derivatization followed a specific injection schedule as follows: 1 µL of the original sample solution was gradually mixed with the solutions in the needle: 5 µL of borate buffer (5 g H_3_BO_3_/100 mL, pH = 11), 3 µL of OPA* reagent, 1 µL of 1 M acetic acid. The separation after derivatization was performed on an XSELECT CSH C18 5 µm column (4.6 × 250 mm; Waters, Milford, MA, USA) equipped with a security guard column (C18, Phenomenex, Torrance, CA, USA) at 30 °C. A mixture of 100 mM Acetate buffer (A; pH 5.8) and HPLC grade Acetonitrile (ACN; B) was used as mobile phase (55:45, *v*/*v*) at a flow rate of 0.4 mL/min; FLD detection was performed at λ_excitation_ = 330 nm and λ_emission_ = 440 nm.

* OPA reagent: 2.5 mg OPA, 400 µL MeOH, 200 µL reducing solution**, 4.4 mL borate buffer.

** Reducing solution: 250 µL 2-mercaptoethanol, 10 mL borate buffer solution.

The standard solutions of AMI (0.5–100 µg/mL) were prepared by diluting the stock solution of AMI (1 mg/mL) with PBS solution (pH = 7.4). An external calibration method was used to determine the amount of the analyte. The limits of detection and quantification were determined as LOD = 0.20 µg/mL and LOQ = 0.60 µg/mL, respectively. The obtained calibration curve was linear with a coefficient of determination R^2^ = 0.9992 in the analyzed concentration ranges.

### 2.13. Loading Efficiency and Encapsulation Efficiency of PLA-Si-Based Nanofibers

The liquid fractions of the samples were also investigated to gauge encapsulation efficiency (EE) and loading efficiency (LE) in percent, as per the following equations (Equation (1): EE; Equation (2): LE):

EE (%) = C_1_ − C_2_/C_2_ × 100 (1)

LE (%) = C_1_ − C_2_/W_m_ × 100 (2)

where C_1_ is the total amount of AMI loading in the nanoparticles (mg), C_2_ denotes the amount of excess AMI in the waste solution after the material had been prepared (mg), and Wm constitutes the weight of the dry mass of the prepared material (mg).

### 2.14. Antibacterial Properties of the AMI-Loaded PLA-Si-Based Nanofibers

Such activity of the AMI-loaded nanofibers against *Staphylococcus aureus* CCM 4516, *Escherichia coli* CCM 4517, *Enterococcus faecalis* CCM 3956, *Klebsiella pmeumoniae* CCM 4415 and *Pseudomonas aeruginosa* CM 1961 was determined by the disk diffusion test (Kirby–Bauer Method), in line with the European Committee on Antimicrobial Susceptibility Testing (EUCAST) [46]. The concentrations of the bacterial suspensions equalled 10^6^–10^7^ cfu/mL. The samples of prepared nanofibers were cut into round pieces (3 mm in diameter, 150 mg (143 ± 27 mg)). The AMI was used in the same quantity. The samples were formed into two piles in one Petri dish, and another inoculated with Mueller–Hinton agar placed on top (performed in triplicate), then the combined plates were incubated at 35 °C for 18 to 24 h. Following incubation, the width of the zone of inhibition for each sample was measured to the nearest millimetre on a SCAN 500 inhibition zone reader (version 8.2.0.0). Nanofibers without AMI were also tested as the control. Each type of fibre was tested in triplicate.

## 3. Results and Discussion

### 3.1. BET Surface Area Analysis and Porosity

**Figure 1:**
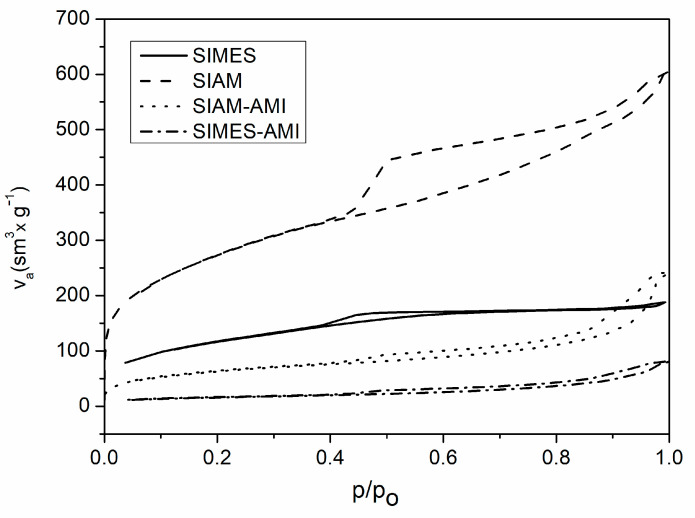
Nitrogen adsorption–desorption isotherms of loaded and non-loaded SIAM and SIMES.

Surface properties were investigated by the BET method. Figure 1 shows the nitrogen adsorption–desorption isotherms of the Si nanoparticles SIAM and SIMES and the AMI-loaded variants SIAM-AMI and SIMES-AMI. The isotherms revealed that they exhibited type IV behaviour typical for mesoporous materials, according to IUPAC classifications [47]. BJH method confirmed the narrow pore size distribution anticipated for this type of material, while mean pore diameters measured 6–8 nm (see Table 3). As detailed therein, the Si nanoparticles SIMES possessed an extremely high surface area and pore volume, brought about by the highly porous Si nanoparticles. The specific surface area decreased significantly after being loaded, as the AMI molecules had taken up some of the available volume, evidencing that the antibiotic had been incorporated within the nanoparticles.

**Figure 2:**
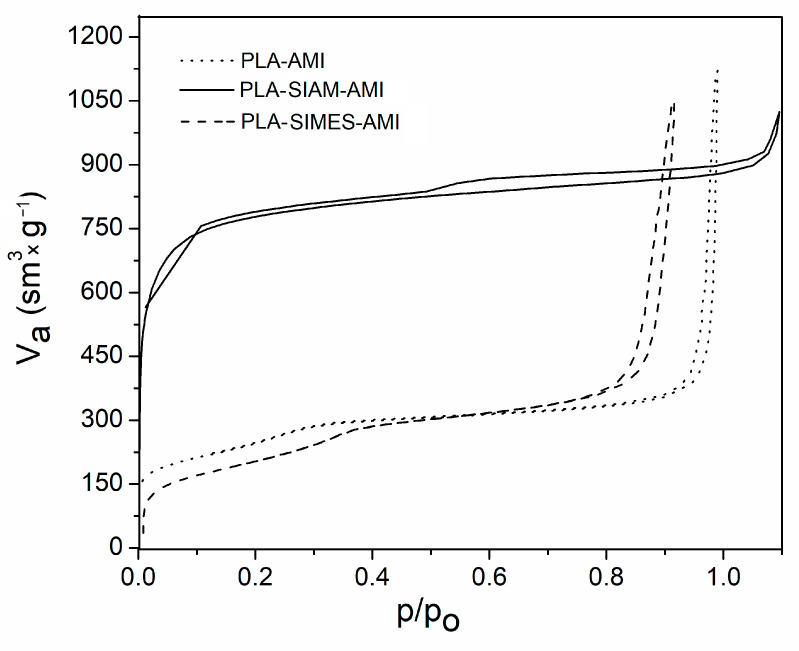
Nitrogen adsorption–desorption isotherms for the electrospun nanofibers.

A standard nitrogen sorption isotherm for the type of PLA-Si-based nanofibers under investigation is given in Figure 2. The other isotherms resemble a type IV curve with H_3_-type hysteresis loops at high relative pressure, according to IUPAC classifications, which is a characteristic of mesoporous materials with great uniformity in size distribution [47,48]. BET analysis of the adsorption–desorption isotherms obtained, in consideration of a sudden jump in adsorption in the p/p_o_ region from 0.8 to 1.0, indicated that the materials possessed a well-defined and regular array of mesopores caused by gaps between the nanofibers. The PLA-SIMES-AMI sample exhibited a hysteresis loop at low relative pressure, connected with the availability of mesopores on the surfaces of the Si nanoparticles, themselves available on the outer sides of the nanofibers. The structured data of each mesoporous material by BET showed that the PLA-Si-based nanofibers had associated high surface areas (see Table 3), large pore volumes, and suitable pore sizes, marking them out as potentially suitable as drug delivery carriers.

### 3.2. Thermal Properties

**Figure 3:**
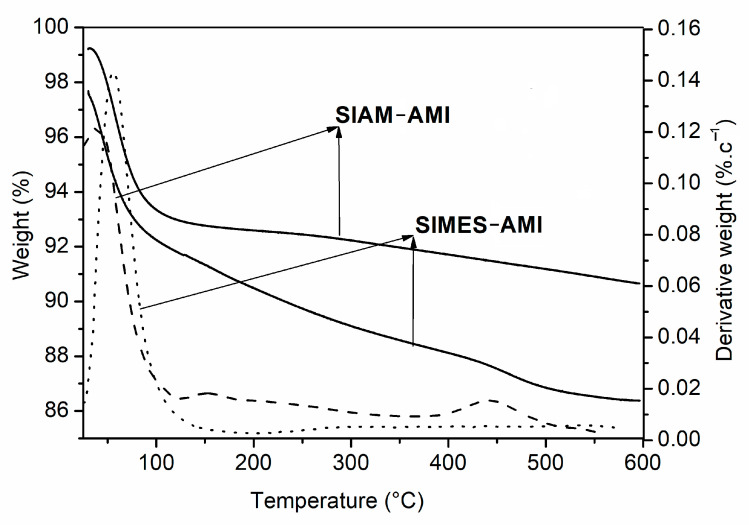
Thermogravimetric profiles of the SIAM-AMI and SIMES-AMI nanoparticles.

Thermogravimetric curves for the Si nanoparticles (SIAM-AMI and SIMES-AMI) are displayed in Figure 3. In line with expectations, the nanoparticles maintained constant mass across the temperature range tested, with the exceptions of an early loss in weight through evaporation of residual water and another from around 100 °C corresponding to degradation. At the final temperature of 600 °C, the SIMES nanoparticles had a residual mass of approximately 87%, and 91% of residual biomass remained for SIAM nanoparticles. Based on these findings, the AMI loaded in Si nanoparticles was calculated as ca 13% and 9% (wt%), respectively.

**Figure 4:**
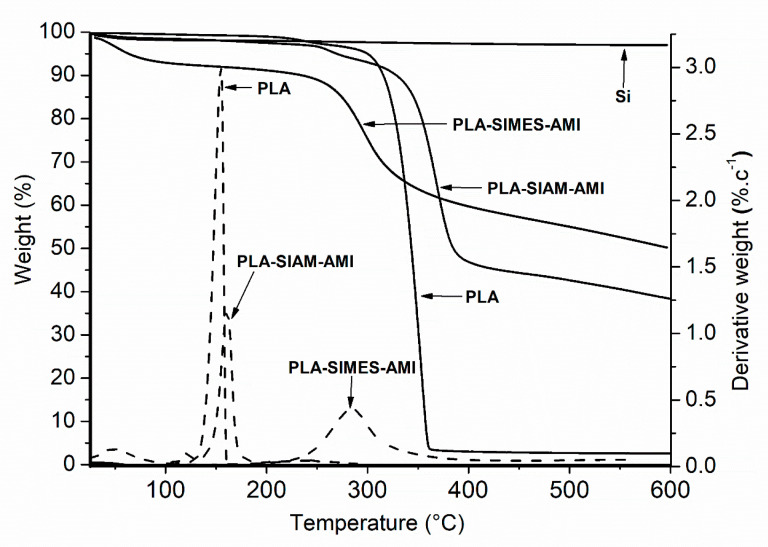
Thermogravimetric profiles of PLA, Si, and AMI-loaded PLA-Si nanofibers- PLA-SIAM-AMI, and PLA-SIMES-AMI.

Figure 4 presents TGA thermograms and dTGA curves for PLA-SIAM-AMI and PLA-SIMES-AMI, alongside those for the PLA nanofibers and Si as references. As expected, PLA-SIAM-AMI and PLA-SIMES-AMI maintained constant mass across the temperature interval of 25–300 °C. An early loss in weight occurred through the evaporation of residual water, and later instances of loss corresponding to degradation started at ca 280 °C for PLA-SIMES-AMI and 350 °C for PLA-SIAM-AMI. At the final temperature of 600 °C, the residual mass of the samples equalled ca 40%, pertaining to the content of Si nanoparticles in the nanofibers. The thermogravimetric profile for the PLA-SIMES-AMI mimicked that for PLA-SIAM-AMI, albeit with a much lower percentage of weight loss due to the greater mass of the Si nanoparticles. Based on these findings, the amount of the Si nanoparticles present in the nanofibers was calculated to equal ca 38% (*w*/*w*) for SIAM and 40% (*w*/*w*) for SIMES, as presented in Table 2.

### 3.3. Loading Efficiency and Encapsulation Efficiency of AMI-Loaded PLA-Si Nanofibers

EE and LE were calculated via Equations (1) and (2), which revealed the Si nanoparticles had a high loading capacity due to their high surface area (Table 4). Values for the loading capacity of the nanofibers were discerned from the remaining AMI left over from the incorporation stage and the encapsulation efficiency of the Si nanoparticles with regard to the resultant nanofibers. This evidenced that the loading capacity of the nanofibers greatly depended on the properties and amount of the Si nanoparticles.

### 3.4. Morphology and Diameter Distribution of the Nanofibers

**Figure 5:**
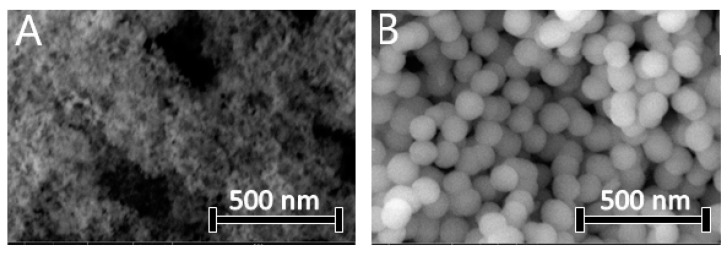
SEM micrographs of (**A**) SIAM-AMI and (**B**) SIMES-AMI nanoparticles.

**Figure 6:**
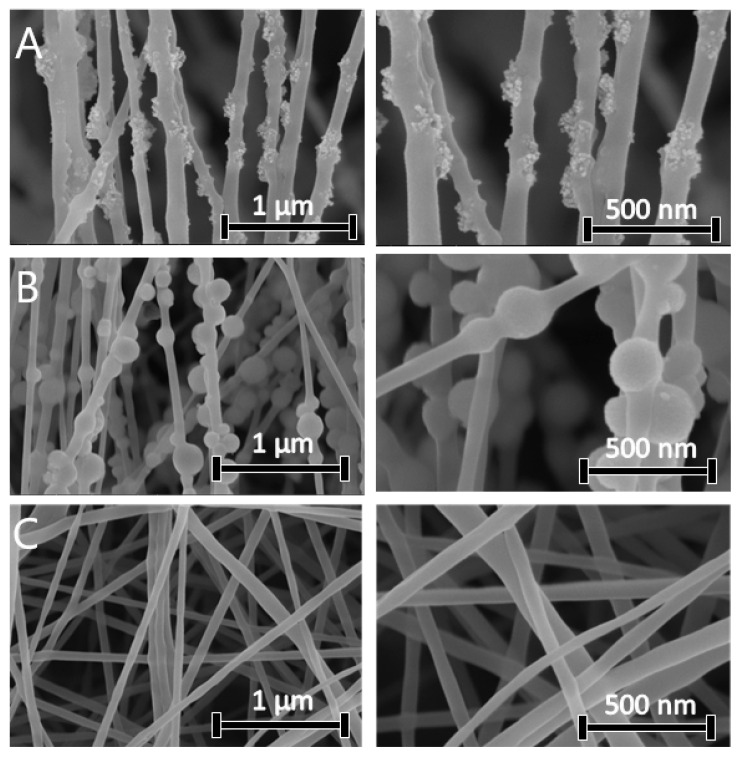
SEM micrographs of the electrospun nanofibers: (**A**) PLA-SIAM-AMI, (**B**) PLA-SIMES-AMI, and (**C**) PLA-AMI.

The morphology of SIAM, SIMES, and electrospun nanofibers was observed by SEM. Figure 5 contains SEM images of the silica nanoparticles applied in the design of the nanofibers. Fumed silica exhibited an aggregated, highly porous structure with a particle size of 30 nm, whereas the mesoporous silica nanoparticles had a spherical morphology and particle size of 200 nm. Figure 6 contains SEM images of AMI-loaded nanofibers. The formulations containing the fumed silica nanoparticles (SIAM) engendered composite nanofibers of a rough morphology. Formulations lacking the Si nanoparticles (PLA-AMI) gave rise to well-formed, bead-free fibres with smooth surfaces that were also the thinnest, with an average diameter of 97 ± 10 nm. They were noticeably thinner (*p* < 0.05) than formulations with Si nanoparticles. The diameter distributions of the AMI-loaded electrospun nanofibers are presented in Table 4. Aggregates of nanoparticles were distributed along the nanofibers, with some clearly visible on the surface. Their diameters were greater, as PLA-SIAM-AMI and PLA-SIMES-AMI measured 135 nm and 150 nm, respectively. In addition, beading affected the outer layer of the polymeric threads. Prior studies state that adding Si nanoparticles into a polymer solution leads to an increase in both viscosity and conductivity, and since these two properties exert opposing effects on the diameters of electrospun nanofibers, supplementing Si nanoparticles in this way can result nanofibers of vast variety of diameters based on the ratio of Si fraction, and suitable parameters could be found for fabrication of nanofibers of optimal diameter compared those fabricated solely from the polymer solution [22,49].

### 3.5. Water Contact Angle

Table 5 details the water contact angles of the electrospun materials. Although some statistical differences existed between the groups of samples, the range of contact angles (93° ± 3) indicated that the pure PLA-AMI nanofibers were hydrophobic in character. A rise in hydrophilic properties depended on the amount of Si nanoparticles with AMI. PLA is a relatively hydrophobic polymer with a water contact angle in the range of 75–85°. Hence, it has a low affinity for cells in biomedical applications and sometimes induces an inflammatory response in surrounding tissue upon direct contact [31]. For this reason, it was supposed that supplementing PLA with silica nanoparticles would change the hydrophobicity of the tested material to a varied extent. A slight decrease in water contact angle for fibres incorporated with Si nanoparticles is reported in the literature [50], supposedly due to the surface presence of hydrophilic silanol groups, a finding verified herein. The nanofibers with the presence of Si were inclined towards a decreased water contact angle. This demonstrated that the Si nanoparticles effectively enhanced the hydrophilic properties of the PLA surfaces. Although there were fewer SIAM nanoparticles than SIMES ones (see Table 2), the hydrophilicity of those samples was greater, probably because of the presence of free silane and silanol groups at a surface level [51].

### 3.6. Elemental Analysis (EDXRF) of the Nanofibers

The EDXRF method was applied to gauge the content of CHO and Si (%) incorporated in the AMI-loaded nanofibers. Taking into consideration the extent of Si present from results obtained for the loading of the Si particles, this method revealed that materials containing SIMES demonstrated a slightly higher presence of Si nanoparticles (see Table 6). As for those loaded with SIAM, a decrease was observed in the amount of Si at 38% when compared to SIMES-loaded nanofibers containing 40% of supplemented nanoparticles.

### 3.7. In Vitro Drug Release Studies

**Figure 7:**
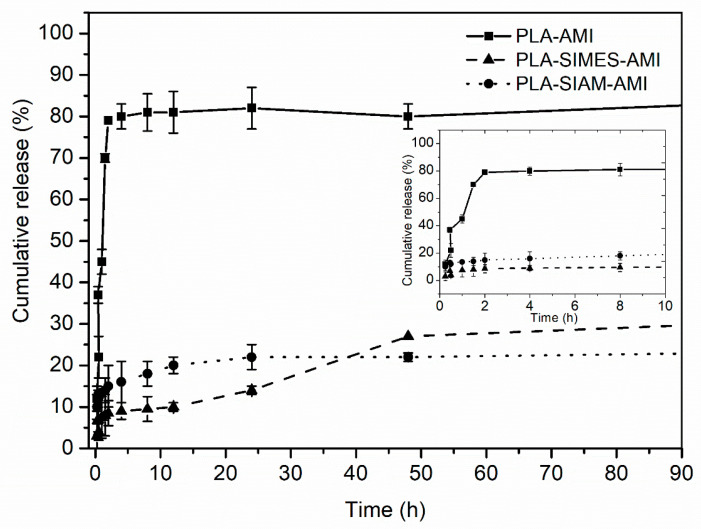
Cumulative release profiles of AMI for the electrospun nanofibers during the first 90 h.

The literature refers to pure PLA composite fibres of a nano/microstructure capable of prolonging the release of several drugs [23,33,52] in studies seeking a means of controlling the release kinetics of AMI. In this study, AMI was immobilized in Si nanoparticles, which in turn were embedded in microsized PLA electrospun nanofibers. Figure 7 presents the in vitro AMI release profiles of the electrospun nanofibers. The first stage in the release profiles of the composite nanofibers involved the release of AMI in SIAM/SIMES-AMI particles immobilized on the surfaces of the nanofibers in direct contact with the release medium and/or the release of AMI embedded directly in the structures of the PLA nanofibers. Secondly, AMI was released from Si nanoparticles embedded inside the nanofibers, requiring penetration of the release medium through the polymer matrix and mesopores, desorption of AMI from the walls of the nanoparticles, and diffusion of AMI out of the mesopores and through the swollen polymer matrix. The explanation for this might lie in the structure of the two composite nanofibers. As mentioned earlier, the PLA-SIAM-AMI nanofibers had an open-pore structure, so when the Si nanoparticles were embedded in the fibrous structure, their surfaces became part of the surface of the fibre, too. The balance between the length of the diffusion pathway and the rate of diffusion across the polymer matrix in each fibre determined the release kinetics. The PLA-AMI exhibited the most rapid release profiles compared to PLA-Si-based nanofibers that showed well-tailored and prolonged release of the drug. The PLA-SIAM-AMI nanofibers exhibited more rapid release kinetics in the first hours of the release study. The gas diffusion pathway was shorter in the PLA-SIMES-AMI nanofibers, heightening the release kinetics and facilitating gradual, steady drug release that clearly transpired in two steps, the latter of them occurring concurrently with the degradation of the nanofibers. The loading of nanoparticles with AMI and their incorporation into a nanofibrous matrix resulted in nanofibers with completely different release profiles from PLA-AMI nanofibers, which achieved almost complete release at the end of the test. The release result of PLA-SIMES-AMI nanofibers is comparable to that of commercial silica nanoparticles SIAM.

### 3.8. Antibacterial Activity Tests

**Figure 8:**
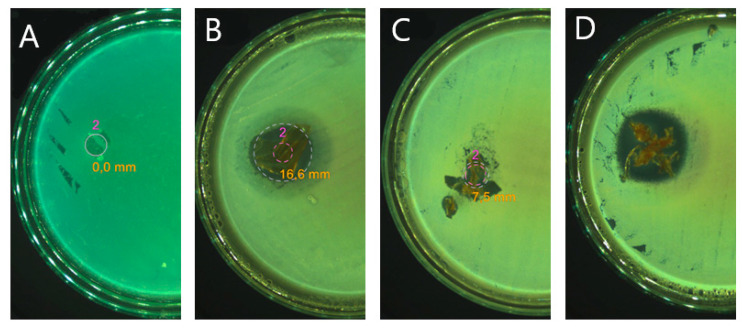
Examples of inhibition zones against *S. aureus*; (**A**) PLA, (**B**) AMI, (**C**) PLA-SIAM-AMI, (**D**) SIAM-AMI.

The fabricated nanofibers were assumed to possess enhanced antibacterial ability against Gram-positive and Gram-negative bacterial strains; hence these properties of the PLA-Si nanofibers were studied in detail. The disk diffusion test was carried out to investigate the antibacterial ability of AMI-loaded fibre materials against selected bacterial strains, which are the most common causative organisms associated with wound infection [52]. As described in Figure 8 below, all the nanofibers containing AMI produced inhibition zones with diameters of between 2 and 8 cm; note that the mean diameter of an inhibition zone was a function of the mean content of AMI in the sample, which was significantly lower than in the reference AMI sample (see Table 2). No inhibition zone was visible around the pure PLA, demonstrating that the observed antibacterial effect was caused by the presence of the AMI. In addition, the presence of silica nanoparticles is likely to contribute to the increased antibacterial effect of AMI. Although the concentration of Si nanoparticles in the nanofibers was lower, it can be seen that SIAM has a greater antibacterial effect compared to SIMES, which could be due to the open-pore structure of the nanofibers that allowed direct contact with the Si surface. Furthermore, if we consider pure AMI, SIAM/SIMES-AMI and PLA-SIAM/SIMES-AMI samples, where less than 5% of the antibiotic was loaded into the carrier itself, only a slight decrease in antibacterial activity was observed, as is clearly visible in Table 7 and Figure 8. In addition, the samples based on fumed silica nanoparticles exhibited a greater antibacterial effect against *S. aureus*, *E. coli*, *P. aeruginosa*, and *K. pneumoniae*, possibly through the more rapid release of the antibiotic from their open-pore structure.

## 4. Conclusions

The aim of this study was to create a novel material with antibacterial properties to counteract wound infection. In this context, two types of AMI-loaded nanoparticles formed of either commercially available fumed silica SIAM or fabricated mesoporous silica SIMES were incorporated in electrospun PLA nanofibers to facilitate prolonged, sustained release of the antibiotic. The results by BET showed that the PLA-Si-based nanofibers had an associated high surface area, large pore volumes, and suitable pore sizes, marking them out as potentially suitable as drug delivery carriers. The incorporation of the silica nanoparticles changed the hydrophobicity of the nanofibers to an extent, which in the context of the intended application, would advantageously curtail inflammatory reactions in living tissue. It was observed that supplementing silica nanoparticles with the AMI and embedding them in nanofibers by electrospinning gave rise to drug-loaded electrospun nanofibers with different release profiles. Experiments revealed that the presence of Si nanoparticles also enhanced the antibacterial properties of the given agent. Since enriching the PLA nanofibers with Si nanoparticles brought about a dramatic increase in antibacterial ability, it would be possible to load the AMI at a limited concentration as a consequence of the Si nanoparticles being so effective against the tested organisms. This antibacterial experiment found that prepared PLA-Si-based nanofibers inherently exerted a strong antibacterial effect against the most common causative Gram-positive and Gram-negative bacteria associated with wound infection, making these nanofibers applicable for wound treatment.

## Figures and Tables

**Table 1:** GPC results for the synthesized PLA.

	*Mw* (g/mol)	*Mn* (g/mol)	*Ð* (-)
PLA	13,800	8000	1.73

**Table 2:** Composition of the electrospun nanofibers.

Formulation	Si (%) ^a^	AMI (%) ^b^	PLA-SIAM/SIMES-AMI (%) ^c^	AMI in Nanofibers (%) ^d^	Si in Nanofibers (%) ^d^
PLA-AMI	-	5	20	1.7	-
PLA-SIAM-AMI	50	5	20	3.9	38
PLA-SIMES-AMI	50	5	20	4.8	40

(^a^) amount of silica nanoparticles, in % (*w*/*w*), in the polymers taken for electrospinning, (^b^) amount of AMI, in %, in electrospinning solution, (^c^) amount of PLA-SIAM/SIMES-AMI, in % (*w*/*v*) in the final electrospinning solution with solvent (chloroform and dimethylformamide), (^d^) Amount of AMI and amount silica nanoparticles, in %, in the resultant nanofibers.

**Table 3:** Values for specific surface area (S_BET_) and pore volume (Vp) for the Si nanoparticles and prepared nanofibers.

Formulation	S_BET_ (m^2^g^−1^)	Vp (cm^2^g^−1^)
SIAM	1200	0.600
SIMES	680	0.50
SIAM-AMI	570	0.180
SIMES-AMI	280	0.307
PLA-SIAM-AMI	1390	1.2
PLA-SIMES-AMI	890	0.804
PLA-AMI	170	178

**Table 4:** EE and LE of the silica nanoparticles.

	EE ± SD ^a^ (%)	LE ± SD ^a^ (%)
SIAM-AMI	66.0 ± 2.0	7.65 ± 2.2
SIMES-AMI	52.0 ± 5.0	12.7 ± 2.3

^a^ standard deviation of three separately prepared samples analysed in triplicate.

**Table 5:** Mean fibre diameters and water contact angles for the electrospun nanofibers. Note: any error bars relating to them were too small to be visible.

Nanofibers	Fibre Diameter (nm)	Water Contact Angle (°)
PLA-SIAM-AMI	150 ± 12	40 ± 4
PLA-SIMES-AMI	135 ± 7	59 ± 5
PLA-AMI	97 ± 10	93 ± 3

**Table 6:** Elemental analysis.

Sample	CHON (%) *	Si (%) *
PLA-SIAM-AMI	62	38
PLA-SIMES-AMI	60	40

* weight in mass percent of basic matrix (% m/m).

**Table 7:** Antibacterial activity of the PLA, AMI, AMI-loaded Si nanoparticles, and fabricated nanofibers using the disk diffusion method.

Width of the Inhibition Zone ± SD ^a^ (mm)					
Sample	*S. aureus*	*E. coli*	*E. faecalis*	*P. aeruginosa*	*K. pneumoniae*
PLA	0	0	0	0	0
AMI	16.5 ± 0.5	16.5 ± 0.5	11	19	18
SIAM-AMI	13	12–13	15	14	12–13
SIMES-AMI	9	9–10	11	12	10
PLA-SIAM-AMI	7.5 ± 0.5	8	0	7	8
PLA-SIMES-AMI	3.5 ± 0.5	3	0	2	5.5 ± 0.5

^a^ SD—Three independently prepared samples, analysed three times.

## Data Availability

The data presented in this study are available in the article.
